# ATP/ADP modulates gp16–pRNA conformational change in the Phi29 DNA packaging motor

**DOI:** 10.1093/nar/gkz692

**Published:** 2019-08-09

**Authors:** Rujie Cai, Ian R Price, Fang Ding, Feifei Wu, Ting Chen, Yunlong Zhang, Guangfeng Liu, Paul J Jardine, Changrui Lu, Ailong Ke

**Affiliations:** 1 Key Laboratory of Science and Technology of Eco-Textiles, Ministry of Education, College of Chemistry, Chemical Engineering and Biotechnology, Donghua University, Shanghai 201620, China; 2 Department of Molecular Biology and Genetics, Cornell University, Ithaca, NY 14853, USA; 3 National Center for Protein Science Shanghai, Shanghai Advanced Research Institute, Chinese Academy of Sciences, Shanghai 201204, China; 4 Department of Diagnostic and Biological Sciences, and Institute for Molecular Virology, University of Minnesota, Minneapolis, MN 55455, USA

## Abstract

Packaging of phage phi29 genome requires the ATPase gp16 and prohead RNA (pRNA). The highly conserved pRNA forms the interface between the connector complex and gp16. Understanding how pRNA interacts with gp16 under packaging conditions can shed light on the molecular mechanism of the packaging motor. Here, we present 3D models of the pRNA–gp16 complex and its conformation change in response to ATP or ADP binding. Using a combination of crystallography, small angle X-ray scattering and chemical probing, we find that the pRNA and gp16 forms a ‘Z’-shaped complex, with gp16 specifically binds to pRNA domain II. The whole complex closes in the presence of ATP, and pRNA domain II rotates open as ATP hydrolyzes, before resetting after ADP is released. Our results suggest that pRNA domain II actively participates in the packaging process.

## INTRODUCTION

**Figure 1.**
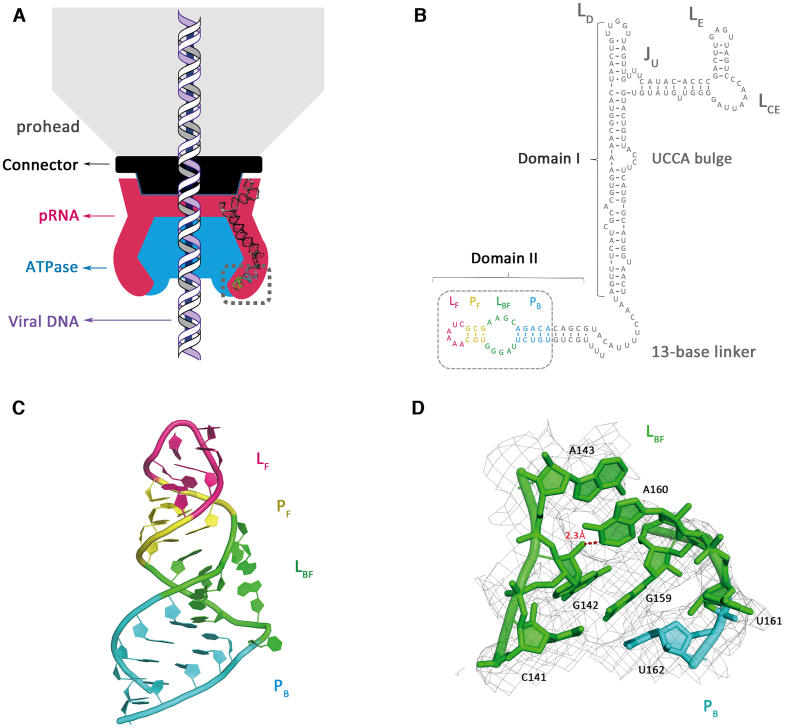
Crystal structure of phi29 pRNA domain II. (**A**) Schematic of bacteriophage phi29 DNA packaging motor. The viral capsid, connector, pRNA and ATPase are shown in gray, black, magenta and blue, respectively. Model of the pRNA domain I was reconstructed with previously published structures (PDB accession codes: 3R4F, 4KZ2). Viral DNA is shown in the middle. Gray dashed box labeled the crystallized pRNA domain II in this study. (**B**) Sequence and secondary structure prediction of the pRNA. The P_B_, L_BF_, P_F_ and L_F_ are colored in cyan, green, yellow and magenta, respectively. The crystallization construct in this study is labeled by the gray dashed box. (**C**) Crystal structure of the pRNA domain II (PDB code: 6JXM). (**D**) A close-up view of the L_BF_ loop superposed on 2Fo-Fc electron density, contoured at 1.0 sigma. N1 of the A160 makes a hydrogen bond to the 2′-OH of G142. Distance is given in angstrom in red.

*Bacillus subtilis* phage phi29 assembles the genome into a preformed protein capsid (prohead) to near-crystalline density by a highly efficient molecular motor (1–3). Studies show that the phi29 DNA packaging motor can generate 57 pN while packing the ∼20 kb genome in under 6 min, making it one of the most powerful motors known, at least 10 times stronger than skeletal myosin (4). The phi29 motor core contains a ribonucleoprotein (RNP) complex of prohead RNA (pRNA) and gene product 16 (gp16) ATPase (Figure 1A) (5). This RNP complex actively transports DNA inside the prohead capsid using energy derived from ATP binding and hydrolysis (6–8). Understanding the mechanism for this molecular motor can provide insights to molecular transport (9), anti-viral therapeutics (10,11) and nanomedicine (12,13).

The phi29 virus and its relatives require a unique, conserved and essential non-coding RNA component, pRNA, to function (14,15). In order to participate in dsDNA packaging, the pRNA forms a pentameric ring via intermolecular base pairing between complementary loops of adjacent RNAs (16,17). Phylogenetic analysis and nuclease digestion studies demonstrated that the phi29 pRNA contains two conserved domains: an 120-base domain I and a 54-base Domain II (18). The previously characterized domain I self-associates into a ring structure via intra-molecular contacts (15,19–21). The resulting ring structure interfaces with the connector protein ring and head shell via a prohead binding subdomain (17,22,23), while the ATPase gp16 binds pRNA via the A-helix subdomain (16). Located near the gp16 ATPase-binding subdomain, the pRNA domain II imparts the specificity and stringency to the packaging process, restricting packaging to only one DNA fragment with fixed orientation in packaging polarity (18,24). However, the molecular mechanism of this highly conserved domain II remained poorly understood as, thus far, it does not appear to participate *in vitro* DNA packaging or force generation.

Similarly, multiple theories were proposed regarding the molecular mechanism for the DNA translocation process. The main powerhouse of the motor, ATPase gp16, provides the drive for packaging using ATP as fuel. Studies show that the N-terminal ATPase domain (M1- S197) binds to the lower end of the pRNA A-stem, while the predicted oligonucleotide/oligosaccharide-binding C-terminal domain (S198- Q332) is located near the ring/three-way junction motif of the pRNA (5,19,21,25–27). Current structural models from crystal structures and mechanistic studies agree that both domains coordinate the step-wise translocation of DNA into the capsid via sequential ATP hydrolysis cycles on each of the gp16 subunits (5,25,28–30). However, no current model illustrates the roles or mechanisms during packaging involving the interactions between gp16 and pRNA with ATP/ADP. This evidence shows the RNP's dynamic and/or transient position and/or function. Thus, more detailed mechanism requires additional structural information on the pRNA–gp16 RNP and its conformation dynamics.

In this study, we investigate the role of pRNA–gp16 RNP in translocating DNA by analyzing its multiple conformations during ADP/ATP binding. First, we solved the pRNA domain II crystal structure at 3.3 Å resolution before docking it into a pRNA–gp16 RNP model derived from the small angle X-ray scattering data. We then isolated three different conformations of the RNP in apo-, ADP- and ATP-bound form, confirmed by both chemical probing and small angle X-ray scattering (SAXS). Our final model shows that gp16 modulates pRNA domain II structural changes induced by ATP and ADP binding. Given the role domain II plays in DNA packaging initiation, these results suggest that ATPase-driven conformational changes in the RNP may participate in the DNA binding event during packaging initiation and perhaps the DNA translocation process as well.

## MATERIALS AND METHODS

### RNA crystal constructs preparation

The pRNA domain II was flanked by a tRNA scaffold as described (31,32) and inserted into the pUC19 plasmid 3′ of a T7 RNA polymerase (RNAP) promoter. The DNA templates for RNA T7 RNAP transcription reaction were prepared by PCR. Then, RNA was purified by urea denaturing gel electrophoresis as described (33,34). The bands corresponding to the pRNA-tRNA hybrid were eluted into RNase-free water at 4°C and refolded by heating up to 65°C for 10 min in a buffer containing 10 mM Na cacodylate pH 7 and 50 mM NaCl, followed by adding 5 mM MgCl_2_ at 65°C for additional 2 min and placed on ice. Resulting samples were concentrated, flash frozen and stored at –80°C.

### RNA crystallization and structure refinement

RNA constructs were screened by hanging drop vapor diffusion at 20°C. The mother liquor contained 0.12 mM spermine, 5 mM cobalt hexamine, 10% (+/−)-2-methyl-2,4-pentanediol (MPD), 62 mM potassium chloride, 30 mM barium chloride, 40 mM pH 6 Na cacodylate. The RNA crystals were grown in drops with 1:1 RNA to mother liquor ratio.

Data were collected at Advanced Photon Source (APS) 24 ID-C Northeastern Collaborative Access Team (NE-CAT) in oscillation mode. Data were processed by HKL-2000 (35). The tRNA–pRNA domain II structure was solved by molecular replacement in Phenix (36), using the tRNA structure (PDB code: 4MGM) (31) as the initial search model. Iterative cycles of building and refining were performed in Coot, Phenix and CCP4 (37–39). Final *R*/*R*_free_ is 22/26.

### ATPase gp16 purification

Wild-type phi29 ATPase gp16 coding sequence was inserted into a pET-28 based SUMO recombinant expression vector. The SUMO-gp16 plasmid was transformed into *Escherichia coli* BL21(DE3) strain and shaken at 200 rpm in LB medium with kanamycin antibiotics at 37°C overnight (24). Then, the culture was diluted 1/100 in fresh LB medium. 1 mM final concentration of isopropyl β-d-1-thiogalactopyranoside (IPTG) was added when OD_600_ reaches 0.5, before lowering the incubation temperature to 18°C for 16 h. The cells were collected and pass through a French press (1000–1200 bar) in a buffer containing 50 mM Tris-HCl, pH 8, 500 mM NaCl, 5% glycerol and 1.5 mM DL-Dithiothreitol (DTT). DNase was added to the lysate at 5 μg/ml and MgCl_2_ at 2.5 mM final. The mixture was incubated at 37°C for 15 min. Then, the lysate was centrifuged at 10 000 rpm for 30 min at 4°C, and the supernatant was loaded onto a pre-equilibrated Ni-NTA column. The column was subsequently washed with 10 column volumes of wash buffer containing 50 mM Tris-HCl, pH 8, 400 mM NaCl, 5% glycerol [vol/vol], 1 mM DTT and eluted with 5 column volumes of elution buffer (50 mM Tris-HCl pH 8, 400 mM NaCl, 5% glycerol, 100 mM imidazole and 1 mM DTT). The elution was concentrated to 100 μl and mixed with 2.5 units of ULP1 protease (Life Technologies) to cleave the SUMO tag. The resulting mix was incubated at 4°C overnight and passed through a Ni-NTA column. The flow through was collected and concentrated in wash buffer.

### RNA preparation and SHAPE probing analysis

The Bacteriophage phi29 174-base wild type pRNA was inserted into a selective 2′-hydroxyl acylation analyzed by primer extension (SHAPE) cassette that contains a 5′ linker, a 3′ linker and a reverse primer binding site and then cloned into the PUC19 vector as described (40). SHAPE probing and data analysis were performed as previously described (17,41–44). The components for prohead-pRNA complex was prepared as described (24,45). The pRNA-free proheads incubated with wild-type 174-base pRNA in 25 mM Tris-HCl, pH 7.5–7.9, 5 mM MgCl_2_, 50 mM NaCl for 15 min at room temperature. Then, the prohead-pRNA were separated by layering on top of 5 ml of 5%(w/v) sucrose and pelleting the proheads through the cushion in the SW55 rotor for 2 h at 35 000 rpm (17). Each sample contained prohead-pRNA, prohead-pRNA-gp16, free pRNA or pRNA-gp16 RNP complex reacted with 0.1 μl NMIA (100 mM in anhydrous DMSO) at 37°C for 45 min. The reaction was quenched with 500 μl of precipitation buffer containing 80% ethanol, 45 μM NaCl, 0.45 μM EDTA (ethylenediaminetetraacetic acid) and 2 μl glycoblue (Ambion). After precipitation at −80°C for 30 min, the RNA was pelleted at 15 000 × *g* for 30 min at 4°C. The pellet was recovered and resuspended in 10 μl of 0.5 × TE. The reverse transcription was similar as described using a FAM labeled DNA primer (FAM-GAACCGGACCGAAGCCCG) (41). Each of reactions and adenosine sequencing ladder was separated by the capillary electrophoresis method used in fragment analysis. Raw traces from fragment analysis were processed by ShapeFinder (42), and SHAPE reactivity differences between pRNA and RNP were identified after normalization and scaling using procedures described previously (43,44).

### Production of pRNA–gp16 RNP complex and packaging intermediates

The pRNA–gp16 RNP complex was reconstituted by refolding WT 174-base pRNA or L_CE_-L_D_ loop mutant in the presence of native gp16 protein as previously described (27,46). Chromatographic separations were performed with the GE AKTA Explorer P100 Chromatography System (GE Healthcare). Then, the mixture was separated by size-exclusion chromatography Superdex 200 Increase 10/300 GL in a running buffer containing 25 mM Tris-HCl, pH 7.5–7.9, 50 mM NaCl, 5 mM MgCl_2_. Stable and homogenous pRNA–gp16 RNP complex was collected and confirmed by SDS-PAGE, Electrophoretic mobility shift assay (EMSA) and Urea-PAGE. To generate the pRNA–gp16 RNP complex with various ligands during DNA packaging process, 0.5 mM ATP/AMP-PNP/ADP were added and incubated for 5 min as described (25,29).

### Electrophoretic mobility shift assay (EMSA)

Free pRNA was incubated with gp16 in 25 mM Tris-HCl, pH 7.5–7.9, 5 mM MgCl_2_, 50 mM NaCl for 15 min at room temperature. The pRNA–gp16 RNP was verified by 0.8% native agarose gel electrophoresis as described (27). The gels were stained with GelStain.

### SAXS data collection and analysis

The small angle X-ray scattering data were collected at the BL19U2 beamline at National Facility for Protein Science Shanghai (NCPSS) and Shanghai Synchrotron Radiation Facility (SSRF). The wavelength was set as 1.033 Å. For SEC-SAXS, 100 μl of ∼7 mg/ml sample was injected on a size-exclusion chromatography Superdex 200 Increase 10/300 GL column equilibrated with 25 mM Tris-HCl, pH 7.5–7.9, 50 mM NaCl, 5 mM MgCl_2_. 2D scattering images were converted to 1D SAXS curves by the software package BioXTAS RAW (47). The matching buffer scattering was subtracted from the sample scattering by PRIMUS (48). All of the preparations were analyzed by linearity in the Guinier region of the scattering data. Pair distribution functions of the particles *P*(*r*) and the maximum sizes *D*_max_ were calculated by the program GNOM (49). Low-resolution shapes were determined from solution scattering data using DAMMIF, from the ATSAS program suite (50,51). Twenty independent calculations were performed by DAMMIF programs for each dataset, using default parameters and no symmetry constraints. Then, 20 independent reconstructions were then averaged and filtered to a final consensus model using the DAMAVER suite (52). Final bead models were visualized by PYMOL (53). Scattering profiles of atomic models were evaluated using FoXS and aligned to the experimental data (54,55). Rigid body modeling was performed using the program SASREF (56). We used SASREF to find relative positions of the pRNA and gp16 by inputting both models separately. Then, we used distance constraints derived from SHAPE and previous EM maps to refine the position of pRNA and gp16 by SASREF. The pentameric models were reconstructed by first fitting the full-length pRNA structure into our SAXS bead model to accommodate all structure motifs, namely the ring domain, three-way-junction, A-stem, linker and domain II. Then, we docked our resulting pRNA model into the previously published cryo-EM and atomic force microscopy structure to reconstruct a pentameric framework (23). Then, the SAXS bead models corresponding to our 3D pRNA model were fitted into the pentameric model.

## RESULTS

### Crystal structure of pRNA domain II

The phi29 pRNA domain II-tRNA crystal structure was solved at 3.3 Å resolution (Figure 1C and Supplementary Table S1) using molecular replacement and multiple rounds of fitting. The secondary structure of pRNA domain II contains two Watson–Crick-paired helices (P_F_ and P_B_) and two loop regions (L_F_ and L_BF_). The crystallization construct contains 31 nucleotides of 3′-domain II, a shortened P_B_ helix and the whole P_F_ helix. Base pairing observed in map density perfectly matches the secondary structure in previous studies (Figure 1B). The pRNA domain II crystal structure shows two double RNA helices (P_F_ and P_B_) connected by a flexible helical turn (L_BF_). The top helix P_F_ is capped by loop L_F_. L_F_ contains four stacked adenines. This loop has the highest thermal factor within the entire structure, with relatively low electron densities, indicating high dynamics and possible tertiary interaction site at L_F_. A second possible flexible region, L_BF_, facilitates a 63-degree turn of the main helix. O2′ of G142 makes an H-bond with N1 of A160 while C141 forms a Watson–Crick basepair with G159, bending the phosphate backbone by ∼60 degrees (Figure 1D). Consequently, U161 flips out to accommodate the turn. A160 and U161 display the highest temperature factors due to their flexibility (Supplementary Figure S2).

### gp16 specifically recognizes pRNA domain II

To investigate the interaction between gp16 and the pRNA and their dynamics, we used selective 2′-hydroxyl acylation analyzed by primer extension (SHAPE) assay to probe the individual nucleotide flexibility of the pRNA.

**Figure 2.**
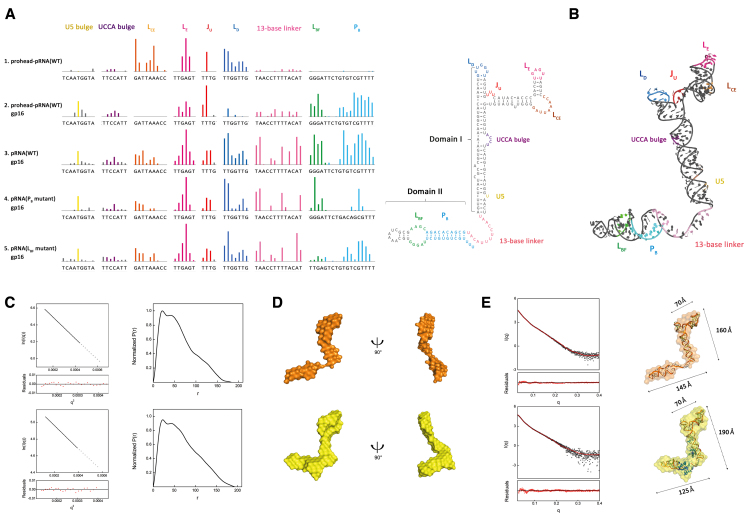
The ATPase gp16 binds to the pRNA and induces conformational change. (**A**) SHAPE analysis shows that gp16 specifically recognizes pRNA domain II. Upward colored bars represent reduced SHAPE activity upon prohead-bound pRNA (row 1), gp16 binding to the prohead-pRNA (row 2) and free wild-type pRNA (row 3), a P_B_ mutant where the sense (UGUCGU) and anti-sense (ACAGCG) strand are switched (row 4) and a L_BF_ mutant (GGGAT to TTGAG) (row 5). Residues are indicated on the *X*-axis. Coloring of pRNA secondary structure is consistent with SHAPE signal. (**B**) Schematic representation of all protected residues on the reconstructed 3D pRNA model. The model contains the previously solved pRNA domain I structure (PDB code: 3R4F, 4KZ2), a helical region grafted from the hexamer pRNA model (PDB code: 1L4O) and a 13-base linker constructed in COOT. Coloring is consistent between the 3D model and SHAPE signal. Protected sites on L_CE_, L_E_, J_U_ and L_D_ of the RNA ring are labeled in orange, magenta, red and navy, respectively, showing intra-RNA interactions. The UCCA bulge and U5 on stem A are shown in purple and yellow, respectively. The 13-base linker is shown in light pink. The signal from L_BF_, P_B_ on pRNA domain II are shown in green and cyan. (**C**) Guinier plot (left) and normalized *P*(*r*) analysis (right) of the pRNA (upper series) pRNA-gp16 RNP (lower series). (**D**) Low-resolution bead models calculated by DAMMIF from SAXS data. The pRNA (upper series) is shown in orange, and RNP is shown in yellow. (**E**) Atomic models of pRNA docked inside the SAXS bead models. The pRNA atomic structure theoretical solution scattering curve (red) is compared to the experimental scattering curves (black) by FoXS. Coloring of pRNA (upper series) and RNP (lower series) bead model is consistent with that in panel (D). Dimensions of RNA ring, stem A and domain II are 70, 160 and 145 Å in pRNA. The three motifs of RNP have dimensions of 70, 190 and 125 Å. The angle between pRNA domain II and A stem is 92°.

First, we determined the gp16 binding influences on the pRNA in both prohead-bound and prohead-free states. Figure 2A demonstrates the protection patterns on the wild-type pRNA complete with prohead and gp16 (row 2), free pRNA with gp16 (row 3), free P_B_ mutant pRNA with gp16 (row 4) and free L_BF_ mutant pRNA with gp16 (row 5), induced by gp16 binding. Both prohead-bound and free pRNA SHAPE probing (rows 2 and 3, also Supplementary Figure S4, rows 2–3) show that gp16 binding stabilizes the overall pRNA tertiary structure with or without prohead. In pRNA domain I, the protection or structural rigidity induced by gp16 binding in both +/− prohead states only differs in L_CE_, L_D,_ L_E_ and the linker region. With prohead bound (row 1, also Supplementary Figure S4, row 1), L_CE_, L_D_ and L_E_ showed increased protection, and these residues correspond to the pRNA ring formation and gp8 binding. After adding gp16, the prohead-pRNA received no additional stability in L_CE_, and L_D_ (row 2), while L_E_ and the linker have gp16-reduced protection compared to the -prohead state (row 3). Rows 3–5 show protection patterns induced by gp16 in prohead-free pRNA in L_D_, J_U_, L_E_ L_CE_ domains, within the three-way junction motif. This motif assembles with the connector proteins to form a ring, channeling the DNA inside. The interactions between L_CE_-L_D_ loop promote the formation of the intermolecular pseudoknot. L_E_ makes direct contact with a unique binding site on the capsid protein (gp8). The elevated protection of these pRNA domains suggests that gp16 binding induced conformational changes in the free pRNA, stabilizing the three-way junction motif. These data indicate that prohead stabilizes L_CE_, L_D,_ L_E_ and linker through the pentameric pRNA quaternary interactions, as gp16 binding does not offer additional protection. Elsewhere, gp16 binding causes a similar stabilization effect in both +/− prohead pRNAs.

Additionally, gp16 binding stabilizes pRNA domain II L_BF_, P_B_ and the flexible linker (Figure 2A, rows 3–5, pink, green and cyan bars). To verify the specific recognition of domain II by gp16, we first mutated the P_B_ sequence with its antisense sequence while maintaining the Watson–Crick base pairing. This P_B_ mutant has reduced binding with gp16 as shown with lower SHAPE signal from protection compared to the wild-type, while other protection patterns were unaffected (Figure 2A row 4, P_B_ mutant). Similarly, replacing the L_BF_ motif from GGGAT to a similar loop from ribosomal RNA (TTGAG) abolishes protection (Figure 2A row 5, L_BF_ mutant). These two mutants demonstrate the sequence-specific interaction between gp16 and the pRNA domain II. Our SHAPE result also shows that pRNA domain II or its binding with gp16 does not affect quaternary interactions in domain I.

### gp16 binding induces conformational change of pRNA

To investigate pRNA conformational change induced by gp16 binding, we performed small angle X-ray scattering (SAXS) on both the wild-type (Supplementary Figure S9) and L_CE_-L_D_ loop mutant of apo pRNA and pRNA–gp16 RNP complex. We speculate the free WT pRNA aggregates upon re-concentration after Size-exclusion chromatography (SEC) (Supplementary Figure S1A), possibly due to the self-assembly nature and base complementarity between L_CE_ and L_D_ in pRNA domain. We have hence mutated L_CE_ and L_D_ in pRNA three-way junction to disrupt any specific interactions without affecting domain II and collected SAXS data with the mutated samples.

Size-exclusion chromatography (SEC) confirmed that the purified pRNA particles bind to gp16, and both the purified pRNA and RNP are monodispersed (Supplementary Figure S1A). Then, the RNP was verified by electrophoretic mobility shift assay (EMSA) in a native agarose gel. Supplementary Figure S1B shows a complete shift in pRNA samples while the RNP retained in the wells. Based on SAXS analysis, the Guinier radius of gyration (*R*_g_) for apo pRNA is ∼43.5 Å, smaller than 52.3 Å of the RNP, calculated from their SAXS profiles (Figure 2C and Supplementary Table S2). Differences between apo pRNA and RNP in the *P*(*r*) distributions occur at the largest interatomic vectors (Supplementary Figure S6). SAXS-based MW estimates put free pRNA at 52 kDa compared to the RNP at 72 kDa. The distinct peaks in Kratky plots of both apo pRNA and RNP indicate that the samples were folded (Supplementary Figure S3).

Our reconstructed 3D models of apo pRNA and RNP (Figure 2B) show that the overall bead model of the pRNA scaffolds resembles an elongated and twisted letter ‘Z’. The three-way junction, L_CE_, L_E_, J_U_ and L_D_ of domain I form the top horizontal stroke, while the linker and domain II form the lower stroke of the ‘Z’, both connected by the A-stem in the middle. We then docked three available structures of the pRNA (PDB 3R4F, 4KZ2 and domain II from this paper) alongside a 21-bp kinked helix (to simulate the stem A of domain I) and a 13-base linker (to simulate the linker region) into our SAXS bead model (Figure 2D and Supplementary Table S3). By fitting and linking those structures, we reconstructed 3D models of the apo pRNA (Figure 2E, upper series) and RNP (Figure 2E, lower series). The resulting 3D model matches the angles and dimensions of the *Z*-shaped bead model. FoXS results show that our pRNA, RNP models agree with the experimental scatter profile (chi^2^ = 2.24 and 1.52, respectively) (Figure 2E). Comparing the apo and RNP 3D bead models, we identified two major differences: (i) the RNP bead model has more volume near the three-way junction and the linker motif between domain I and II; (ii) the linker and domain II of the RNP extend and swing further out of the plane and upwards compared with the apo model. Both changes attribute toward the binding of gp16.

The extra molecular mass from SAXS allowed us to position the gp16-binding sites at the three-way junction and domain II plus linker motif (Figure 2E). This binding position directly supports our SHAPE probing and mutagenesis experiment. Additionally, both our SAXS and SHAPE data showed that gp16 binding compacts and stabilizes the pRNA. Our fitted 3D model shows that gp16 binding compresses the domain II plus linker arm (Figure 2D). The lengths of the apo pRNA ring, A-stem helix and domain II measure 70, 160 and 145 Å, respectively (Figure 2E, upper series), while those of the RNP measure 70, 190 and 125 Å (Figure 2E, lower series). Overall, our experiments show that gp16 binds to the pRNA at two specific locations and alters the conformation and flexibility of the pRNA.

### ATP and ADP induce rotations in pRNA domain II

To investigate how gp16 modulates pRNA conformational changes, we performed SHAPE and SAXS experiments on the RNP in the presence of ATP, gamma-s-ATP, AMPPNP and ADP. Since both SHAPE and SAXS results show identical results for pRNA-gp16+ATP and +AMPPNP, we opt to combine +ATP and +AMPPNP results herein.

**Figure 3.**
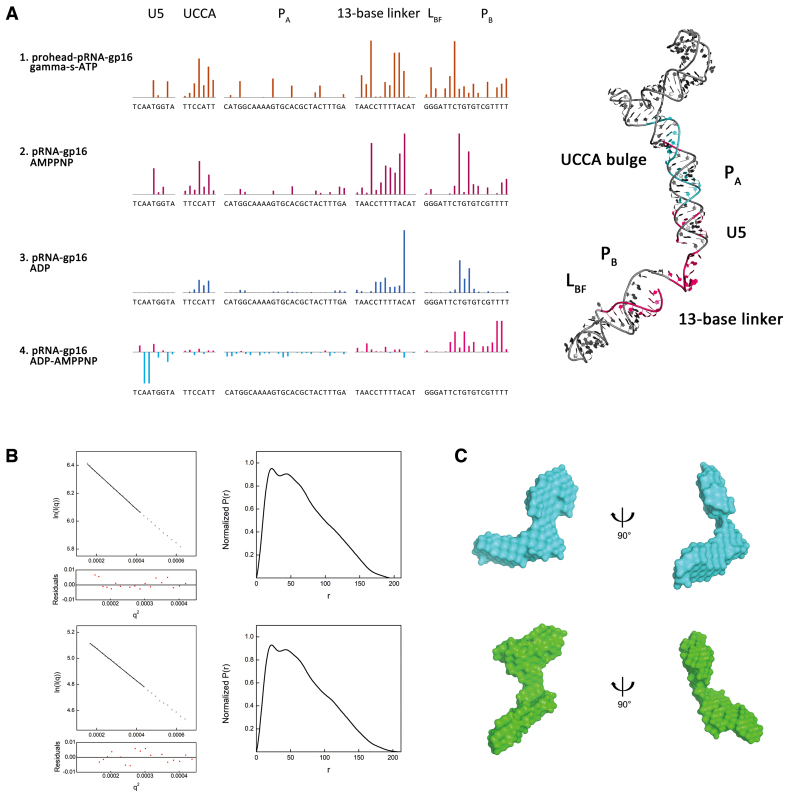
ATP and ADP modulate the pRNA-gp16 RNP conformational change. (**A**) Quantitative analysis of the RNP SHAPE data reveals that the ATP and ADP induce two different pRNA conformations. Row 1 brown bars represent protected residues caused by ATP binding in prohead–pRNA–gp16, namely UCCA-bulge, the 13-base linker and P_B_ in domain II. Row 2 purple bars show the ATP protection sites in pRNA–gp16. Row 3 indicates protected residues due to ADP binding. Row 4 shows the differential protection pattern between ATP and ADP. Positive magenta bars represent the ATP-induced protection relative to ADP binding. Right panel: protected residues mapped on the 3D structure of full-length pRNA structure. Coloring is consistent across all panels. Residues are indicated on the *X*-axis. (**B**) Guinier plot (left) and normalized *P*(*r*) analysis (right) of the ATP-bound RNP (upper series) ADP-bound RNP (lower series). (**C**) SAXS bead models of the ATP-bound (cyan, upper) and ADP-bound (green, lower) RNP.

Figure 3A shows similar domain movement induced by ATP binding in the wild-type pRNA complete with prohead and gp16 (row 1) and free pRNA with gp16 (row 2). Overall, the wild-type motor complex responds more to ATP binding by having higher ATP-induced protection than the free pRNA–gp16 RNP (Supplementary Figure S4, rows 4–5). Both +/− prohead probing data produce similar protection signals attributed to domain II movement in P_B_, U5 bulge, UCCA bulge and the 13-base linker. These data indicate that ATP induces pRNA–gp16 conformation change independent of prohead binding.

Figure 3A rows 2–3 reveal shifts in pRNA backbone dynamics between ATP and ADP binding. The probing patterns between apo and ATP/ADP-bound suggest that both ATP and ADP induces elevated protection in the linker and domain II area. ATP/ADP binding stabilizes the 13-base linker region, indicating that domain II maintains its relative positions in both states. However, comparing the data between ADP and ATP reveals that they induce two distinct conformations of pRNA (Figure 3A, row 4).

To visualize the multiple conformations of the pRNA–gp16 RNP induced by ATP and ADP, we calculated their respective molecular bead model using SAXS profiles (Figure 3B and C; Supplementary Figure S3 and Supplementary Table S3). Both ATP and ADP bound structures show similar architecture at the three-way junction, ring domain and A-stem. However, ADP causes the linker, domain II and N-terminal gp16 region (the lower arm) to swing outward from the DNA channel by 93 degrees, while ATP brings the lower arm close to the DNA channel, forming a second ring-like structure at the bottom of the motor complex. Both RNP models (ADP/ATP) fit the experimental scattering curves, yielding chi^2^ = 1.87, 1.77, respectively (Supplementary Figure S5A). Comparing real space *P*(*r*)-distributions of ADP-bound, ATP-bound and apo-RNP, the ADP-bound form appears extended while the ATP-bound pRNA revealed small but noticeable differences (Supplementary Figure S7A and B). Meanwhile, the Log-Log plot of ATP-bound RNP and ADP-bound RNP showed conformation differences (Supplementary Figure S7C). Hence, we conclude that ADP, not ATP, induces large domain II movement in pRNA. Then, we reconstructed the pRNA–gp16 RNP with ATP/ADP by SASREF (Supplementary Figure S5B). The final model agrees with our SAXS bead-model.

**Figure 4.**
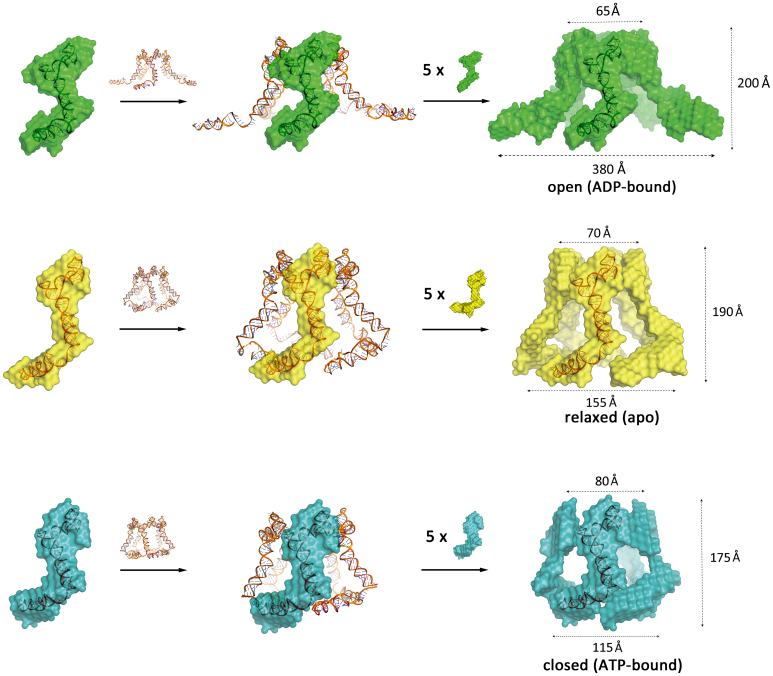
Reconstructed pentameric 3D models of ADP-, apo- and ATP-bound RNP. The full-length pRNA structure was docked into previously published cryo-EM and atomic force microscopy structure to reconstruct a pentameric framework (23). Then the ADP–bound RNP SAXS bead model (green), RNP SAXS bead model (yellow) and ATP–bound RNP SAXS bead model (cyan) were fitted onto the pentameric framework. The top inner pore, bottom inner pore and height of the ‘open’ model are ∼65, 380 and 200 Å. The top inner pore, bottom inner pore and height of RNP pentameric model are ∼70, 155 and 190 Å. The top inner pore, bottom inner pore and height of the ATP–bound RNP pentameric model are ∼80, 115 and 175 Å. The lower arm angle difference between the ATP and ADP-bound complex is ∼93°.

Next, we reassembled the individual pRNA–gp16 pair into a ring structure, based on known 3D models of the pRNA–gp16–prohead complex (Figure 4 and Supplementary Figure S8). We first manually docked our full-length atomic pRNA model into previously published cryo-EM and atomic force microscopy structure to reconstructed a pentameric wire framework (23). Then, our SAXS bead models corresponding to our 3D pRNA model were drawn onto the pRNA wire frame to form the pentameric ring. This pentameric RNP (apo) forms a compact barrel-like structure with a central channel with dimensions measuring 190 Å deep, 70 Å wide at the top where it binds to the prohead ring and 155 Å wide at the bottom where DNA is threaded through. Figure 4 (lower series) models show that ATP forms a compact RNP complex, with a 80 Å top opening, 115 Å bottom opening and 175 Å barrel height. The ADP-bound structure forms an ‘open’ complex, with a slightly constricted top opening at 65 Å, a channel height of 200 Å and a wide bottom channel of 380 Å. During the translocation of DNA, each RNP cycles through all three conformations. The ADP-bound RNP does not associate with the DNA, and upon the release of ADP, the lower arm resets to a neutral position. Conversely, upon ATP binding, the lower arm contracts and locks with the DNA to facilitate its transfer upward into the capsid. During this process, the ring and A-stem stay relatively stationary across all three conformations, with the only difference observed was the location and angle of the lower arm.

In conclusion, we described the structure and dynamics of phi29 DNA packaging complex involving wild-type packaging motor gp16 and pRNA. First, we presented the 3.3 Å crystal structure of the pRNA domain II detailing its atomic structure. Next, we identified gp16–pRNA interactions by SHAPE chemical probing. Subsequent SAXS experiments revealed molecular bead models of different gp16–pRNA conformations, supported by both the crystal structure and SHAPE data. Our combined result characterizes three distinct conformations of the gp16–pRNA conformation: apo-, ADP-bound and ATP-bound. Our models indicate that gp16 binds to the pRNA and manipulates pRNA domain II to facilitate the movement and specific recognition of DNA by cycling through these conformations.

## DISCUSSION

In our study, we investigated the pRNA–gp16 complex structure through crystallography, SAXS and chemical probing. Our data shows that the RNP resembles a twisted letter ‘Z’ and exists as a stable RNP complex in solution. From our data, gp16 contains two distinct structural domains that interact with A-stem and domain II separately, consistent with previous studies (27). By comparing the SAXS bead models between pRNA and pRNA–gp16 RNP, our data support the previous result that the C-terminal domain of gp16 binds to the three-way junction and the N-terminal domain positions near the pRNA domain II (25). Our data shows that motifs (L_D_, L_E_ L_CE_ and C, E, D helices) that bind prohead and connector proteins have little conformational change upon gp16 binding. This result agrees with previous findings that the pRNA assembles with the prohead independent of gp16 (15,57,58). Additionally, our prohead-free RNP responded to ATP/ADP binding similarly as the wild-type prohead-bound RNP, indicating that prohead does not influence the ATP/ADP-induced domain II behavior. According to our SHAPE and SAXS data above, our RNP exhibits active ATPase functions independent of reassembly, similar to previous studies (46,59). This evidence provides biological relevance to our structural and functional studies to investigate the function of pRNA domain II and its interaction with gp16.

Our crystal structure, mutagenesis, chemical probing and SAXS data suggest that the pRNA domain II serves as an extension to the gp16. The ATPase gp16 actively, specifically recognizes and manipulates the pRNA domain II during the packaging process. The domain II consists of two conserved and unique internal loops, L_F_ and L_BF_, both displaying high temperature factors and relatively diffused electron densities. The remaining helices, P_B_ and L_BF_, are recognized by ATPase gp16. This layout suggests that these conserved internal structures may interact with other viral components (such as gp3) as suggested previously (24,60).

**Figure 5.**
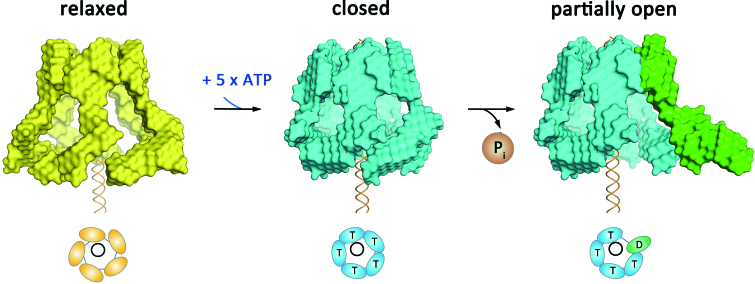
Reconstructed 3D models of the phi29 molecular movement during ATP binding and hydrolysis. During ATP binding, the individual RNP switches from the ‘relaxed’ conformation (yellow bead models) to the ‘closed’ conformation (blue bead model labeled with ‘T’s). Subsequent ATP hydrolysis opens the complex (green subunit labeled with ‘D’) by swinging the pRNA domain II outwards.

Our structural model suggests that ATP binding to gp16 induces a closed motor and DNA contact, while its subsequent hydrolysis may release DNA and open up the complex (Figure 5). Little is known about the process that anchors the DNA–gp3 substrate onto the motor prior to translocation. Given that domain II conveys the selective property of recruiting only the left end of the otherwise symmetrical viral DNA, the dynamics demonstrated here likely play a direct role in this process by engaging a conformational switch that loads the DNA into the translocating channel, implying a nucleotide dependent process. Importantly, domain II has never been visualized using cryo-EM 3D reconstruction in either proheads alone or in the intact translocation complex, implying a transient role of domain II in motor assembly.

What remains to be determined is whether domain II plays an active role in DNA translocation. Given that the gp16–pRNA ribonucleotide complex changes conformation under the influence of the ATP binding and hydrolysis cycle, we may infer that the movement of domain II plays an active role in DNA movement. The DNA packaging process involves gp3 binding to DNA, with subsequent association and packaging by the assembled prohead. The terminal protein gp3, essential for DNA packaging, covalently attaches to the first adenine of each 5′ ends of the phi29 genome as a part of the protein-primed replication strategy adopted by these phages. During the packaging process, pRNA–gp16 directly interacts specifically with DNA–gp3 (21,60). Although currently no studies reported direct interaction between gp3 and pRNA domain II, post-packaging EM image puts gp3 near where domain II would have located prior to motor disassembly (61). We therefore believe domain II conformation change would affect gp3–DNA complex binding to the packaging motor, affecting the initiation process. This observation may result in directional DNA recognition that leads to packaging polarity. Whereas no data suggest the need for domain II in extensive *in vitro* studies in the packaging process, any conclusion regarding domain II function still requires *in vivo* comparison between the full-length pRNA and a truncated domain I variant . A more direct comparison would determine a direct role for domain II, if any, in the force generating mechanism of DNA packaging. Given the ordered nature of the nucleotide-dependent conformational changes we observe in the pRNA–gp16 complex, we reveal that this interaction to be dynamic and likely reflect transient packaging initiation events that are not well understood in any DNA virus system. These critical events to virus assembly provide a novel target for therapeutic studies.

## DATA AVAILABILITY

Data deposition: The coordinates for the pRNA domain II structure have been deposited in the Protein Data Bank, www.pdb.org (PDB ID code 6JXM).

## Supplementary Material

gkz692_Supplemental_File
