# Supplementary material for: ATP/ADP modulates gp16–pRNA conformational change in the Phi29 DNA packaging motor
Source: Nucleic Acids Res. 2019 Aug 9;47(18):9818–28. doi: 10.1093/nar/gkz692 (PMC6765105; doi:10.1093/nar/gkz692)
Supplement: gkz692_Supplemental_File [file gkz692_supplemental_file.pdf]

Supplementary Information for

ATP/ADP Modulates gp16-pRNA Conformational Change in The Phi29 DNA Packaging Motor

Rujie Cai[1], Ian R. Price[2], Fang Ding[2], Feifei Wu[1], Ting Chen[1], Yunlong Zhang[1], Guangfeng Liu[3], Paul J. Jardine[4], Changrui Lu[1]*, Ailong Ke[2]*

[1]Key Laboratory of Science and Technology of Eco-Textiles, Ministry of Education, College of Chemistry, Chemical Engineering and Biotechnology, Donghua University, Shanghai, 201620, China;

[2]Department of Molecular Biology and Genetics, Cornell University, Ithaca, NY, 14853, USA;

[3]National Center for Protein Science Shanghai, Shanghai Advanced Research Institute, Chinese Academy of Sciences, Shanghai 201204, China;

[4]Department of Diagnostic and Biological Sciences, and Institute for Molecular Virology, University of Minnesota, Minneapolis, MN 55455, USA;

*To whom correspondence should be addressed. Tel: 86-21-67792740; (607) 255-3945. Fax: (607) 255-6249
E-mails: crlu@dhu.edu.cn; ak425@cornell.edu.

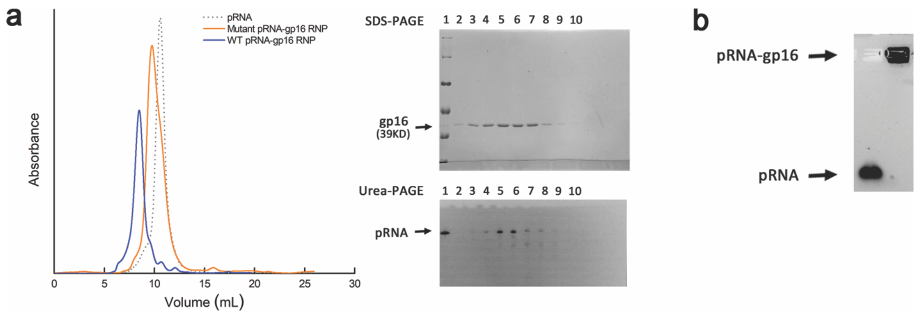

Supplemental Figure 1. pRNA and gp16 interaction in solution**.** (a) Size exclusion chromatographic (SEC) analysis of wild type pRNA-gp16 RNP and mutant pRNA-gp16 RNP. Mutant pRNA-gp16 RNP complex was collected and confirmed by SDS-PAGE and Urea-PAGE (shown with arrow). The SDS-PAGE lane 1: protein marker; lanes 2-10: peak fractions. The Urea-PAGE lane 1: 174-base pRNA as compared; lanes 2-10: peak fractions. (b) EMSA of pRNA incubated with gp16.

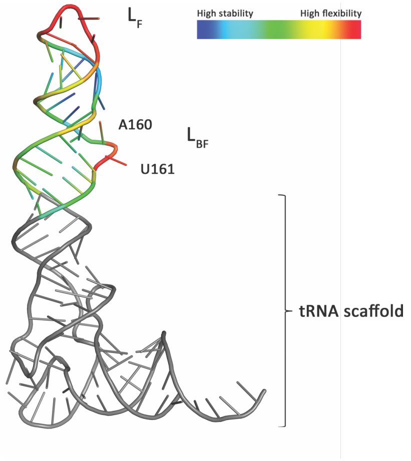

Supplemental Figure 2. B factor analysis shows the flexibility of the pRNA domain II. Individual nucleotides are colored from blue to red, based on their temperature B factors ranging from 100 to 240. Significantly higher B factors at the L_F, unpaired A160 and U161 residues in L_{BF} are shown in red. tRNA scaffold is shown in grey.

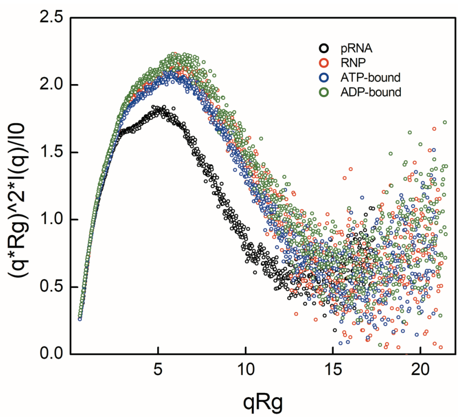

Supplemental Figure 3. Dimensionless Kratky plots. pRNA (black), RNP (red), ATP-bound (blue) and ADP-bound (green) pRNA-gp16 RNP show a typical peak at low scattering angle and then return to baseline, suggesting that all particles are in the native state.

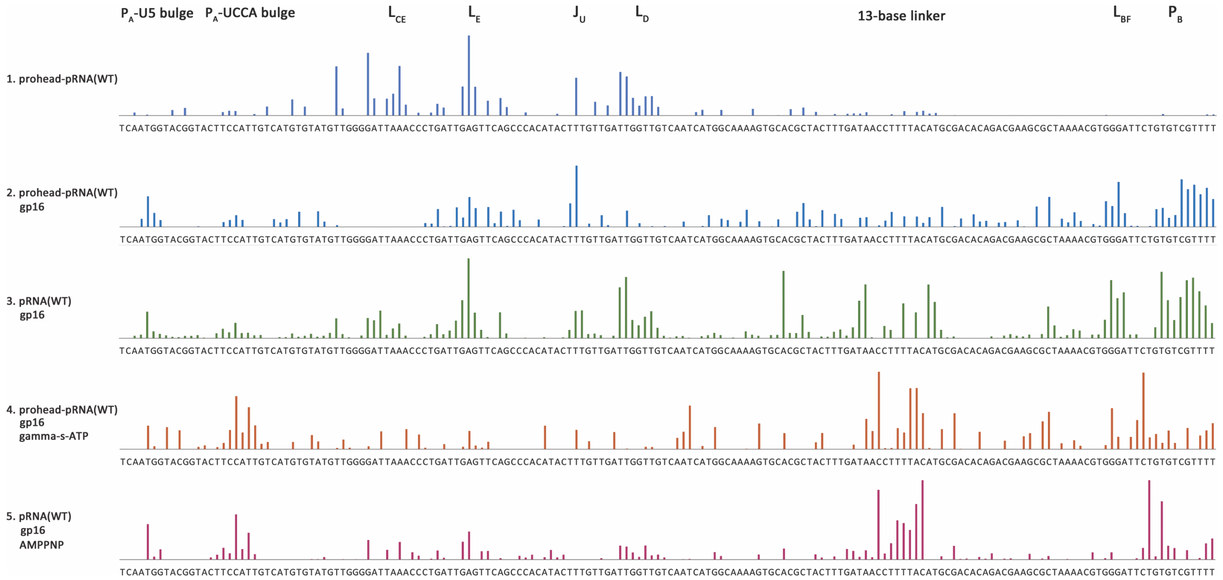

Supplemental Figure 4. SHAPE analysis shows the ATPase gp16 binding sites and the pRNA-gp16 RNP conformational change modulating by ATP. Positive bars indicate structural rigidity. Row 1 shows protection by prohead binding. Row 2 row 3 bars represent protection by gp16 binding to prohead-pRNA and free wild-type pRNA. Row 4 shows protection caused by gamma-s-ATP binding to prohead-pRNA-gp16 complex. Row 5 represents the AMPPNP-induced protection in free wild-type pRNA-gp16 RNP.

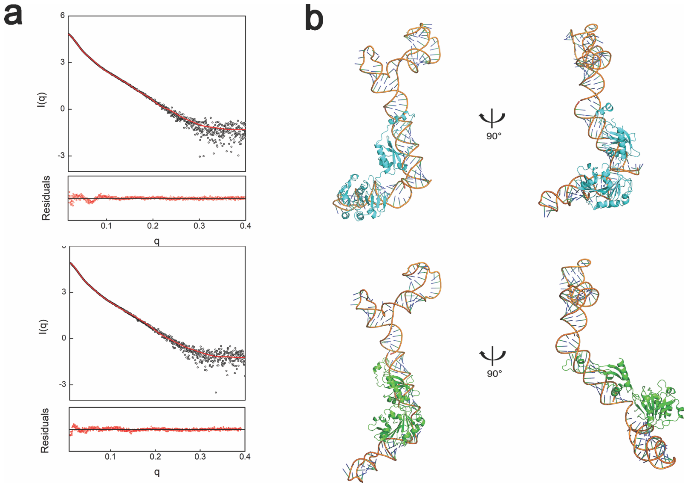

Supplemental Figure 5. (a) FoXS of the ATP-bound RNP (upper) and ADP-bound RNP (lower). (b) SASREF model of ATP-bound RNP (upper) and ADP-bound RNP (lower).

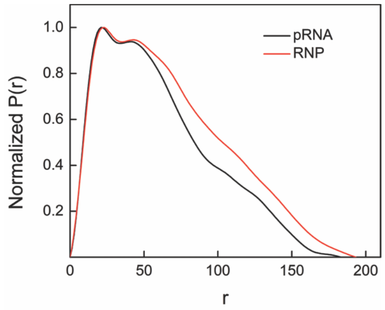

Supplemental Figure 6. Normalized P(r) analysis. Compare the properties of the pRNA (black) and pRNA-gp16 RNP (red).

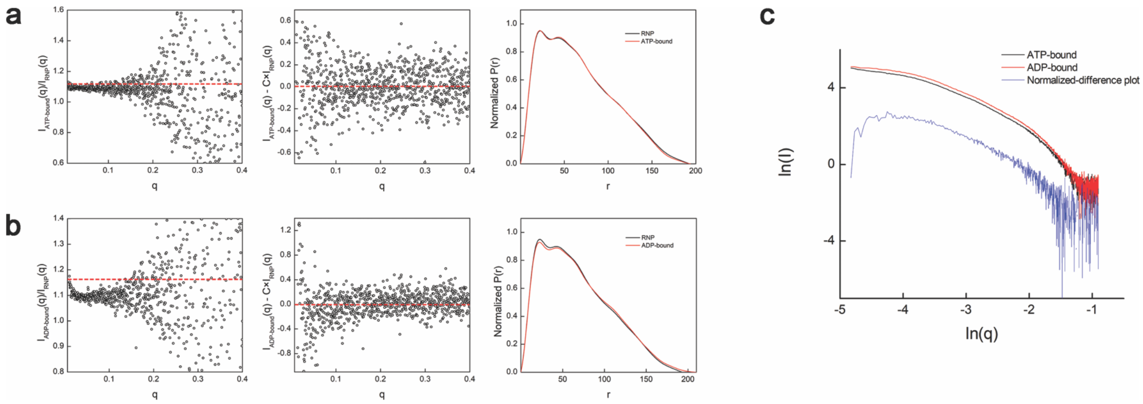

Supplemental Figure 7. Scattering curve comparisons between ADP-bound/ ATP-bound with apo RNP. (a) Difference plot of ATP-bound RNP with apo RNP. (b) Difference plot of ADP-bound RNP with apo RNP. (c) Log-Log plot analysis of ATP-bound RNP (black) and ADP-bound RNP (red).

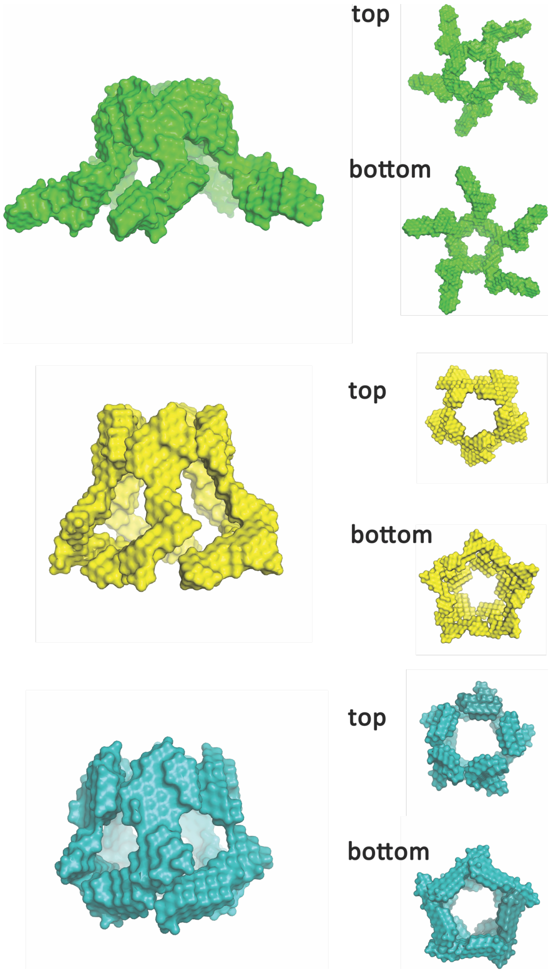

Supplemental Figure 8. Top-down image of pentameric 3D models of ADP-bound RNP (green), apo RNP (yellow) and ATP-bound RNP (cyan).

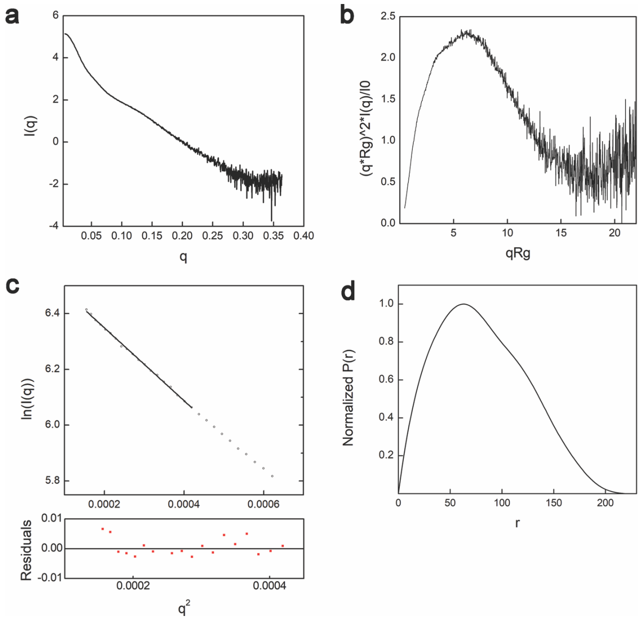

Supplemental Figure 9. SAXS curves of WT pRNA-gp16 RNP. (a) Scattering curve. (b) Dimensionless Kratky plots. (c) Guinier plot (d) Normalized P(r) analysis.

Supplemental Table 1. Crystallographic statistics

	pRNA domain II- tRNA cobalt Hex
**Data Collection**	
Beamline	APS 24 ID-C
Wavelength (Å)	1.606
Space group	I422
Unit cell (a, b, c)	120.18, 120.18, 187.49
Resolution range (Å)	101.18-3.32
Multiplicity	8.5
Completeness (%)	98.2
Mean I/sigma(I)	13.9
Wilson B-factor (Å^2)	160.7
R-merge	0.106
R-meas(I)	0.114
Rpim(I)	0.041
Total observations	89650
Unique reflections	10506
**Refinement**	
Resolution (Å)	3.3
Total non-hydrogen atoms	2077
Number of Unique reflections refined against	9296
RMS (bonds, Å)	0.49
RMS (angles,°)	0.72
Clash score	6
R_{work}/R_{free}	0.223/ 0.265
Average B-factor (Å^2)	162

Supplemental Table 2. SAXS sample details and parameters

	pRNA	RNP	AMP-PNP	ADP
SEC-SAXS column	Superdex 200 Increase 10/300 GL			
Loading concentration (mg/ml^{-1})	7.5	6.5	6.8	7
Injection volume (µl)	100	100	100	100
Flow rate (ml/min^{-1})	0.3	0.3	0.3	0.3
Solvent	25 mM Tris-HCl pH 7.5~7.9, 50 mM Nacl, 5mM $Mgcl_2$			
**Guinier analysis**				
qR_g max	1.2	1.24	1.22	1.29
R_g (Å)	43.50± 0.13	52.3 ± 1.4	51.35± 0.37	50.91 ± 0.48
I(0) (cm^{-1})	120.38 ± 0.64	126.53 ± 2.45	136.10 ± 1.6	137.94 ± 1.19
**GNOM**				
R_g (Å)	49.01	54.46	54.26	56.04
I(0) (cm^{-1})	128.2	127.5	139.2	143.7
D_{max} (Å)	183.15	196.84	194.43	210.45
q range (Å^{-1})	0.009-0.255	0.008-0.262	0.009-0.248	0.009-0.275

Supplemental Table 3. NSD metric for the averaging of SAXS model

pRNA	Aver	1	2	3	4	5	6	7	8	9	10	11	12	13	14	15	16	17	18	19	20
1	0.90	0.00	1.06	0.96	0.85	1.00	0.78	0.89	0.99	0.92	0.91	0.83	0.92	0.94	0.92	0.77	0.93	0.88	1.00	0.89	0.75
2	0.93	1.06	0.00	1.06	0.96	0.87	0.93	0.94	0.99	0.85	0.84	1.00	0.86	0.96	0.88	0.92	0.85	0.95	0.90	0.85	1.00
3	0.96	0.96	1.06	0.00	0.99	0.96	0.93	0.93	1.02	0.82	0.98	1.00	0.82	0.98	1.09	0.98	1.01	0.91	0.93	1.00	0.89
4	0.89	0.85	0.96	0.99	0.00	0.86	0.93	0.85	0.89	0.83	0.94	0.73	0.91	0.83	1.04	0.78	0.91	0.81	0.86	1.01	0.90
5	0.91	1.00	0.87	0.96	0.86	0.00	0.99	0.91	0.87	0.91	0.99	0.88	0.88	0.94	1.02	0.96	0.70	0.92	0.85	0.86	0.94
6	0.87	0.78	0.93	0.93	0.93	0.99	0.00	0.87	0.82	0.96	0.67	0.94	0.94	0.70	0.81	0.86	1.02	0.94	0.88	0.81	0.75
7	0.86	0.89	0.94	0.93	0.85	0.91	0.87	0.00	0.87	0.72	0.94	0.90	0.84	0.77	0.83	0.80	0.83	0.81	0.86	0.84	0.92
8	0.89	0.99	0.99	1.02	0.89	0.87	0.82	0.87	0.00	0.83	0.92	0.87	0.77	0.85	0.82	1.02	0.97	0.85	1.01	0.70	0.87
9	0.87	0.92	0.85	0.82	0.83	0.91	0.96	0.72	0.83	0.00	0.95	0.84	0.71	0.93	0.89	0.91	0.96	0.86	0.77	0.89	0.93
10	0.89	0.91	0.84	0.98	0.94	0.99	0.67	0.94	0.92	0.95	0.00	1.03	0.92	0.90	0.66	0.92	0.99	0.93	0.88	0.80	0.81
11	0.88	0.83	1.00	1.00	0.73	0.88	0.94	0.90	0.87	0.84	1.03	0.00	0.90	0.80	0.98	0.86	0.81	0.75	0.88	0.88	0.75
12	0.87	0.92	0.86	0.82	0.91	0.88	0.94	0.84	0.77	0.71	0.92	0.90	0.00	0.96	0.79	0.90	0.90	0.89	0.87	0.80	0.92
13	0.88	0.94	0.96	0.98	0.83	0.94	0.70	0.77	0.85	0.93	0.90	0.80	0.96	0.00	1.03	0.73	0.82	0.98	0.95	0.85	0.75
14	0.90	0.92	0.88	1.09	1.04	1.02	0.81	0.83	0.82	0.89	0.66	0.98	0.79	1.03	0.00	1.07	0.97	0.72	1.02	0.75	0.86
15	0.88	0.77	0.92	0.98	0.78	0.96	0.86	0.80	1.02	0.91	0.92	0.86	0.90	0.73	1.07	0.00	0.80	0.84	0.86	0.98	0.84
16	0.90	0.93	0.85	1.01	0.91	0.70	1.02	0.83	0.97	0.96	0.99	0.81	0.90	0.82	0.97	0.80	0.00	0.79	0.93	0.94	0.92
17	0.87	0.88	0.95	0.91	0.81	0.92	0.94	0.81	0.85	0.86	0.93	0.75	0.89	0.98	0.72	0.84	0.79	0.00	0.88	0.85	0.96
18	0.91	1.00	0.90	0.93	0.86	0.85	0.88	0.86	1.01	0.77	0.88	0.88	0.87	0.95	1.02	0.86	0.93	0.88	0.00	1.04	0.91
19	0.87	0.89	0.85	1.00	1.01	0.86	0.81	0.84	0.70	0.89	0.80	0.88	0.80	0.85	0.75	0.98	0.94	0.85	1.04	0.00	0.83
20	0.87	0.75	1.00	0.89	0.90	0.94	0.75	0.92	0.87	0.93	0.81	0.75	0.92	0.75	0.86	0.84	0.92	0.96	0.91	0.83	0.00
Aver	0.89	0.90	0.93	0.96	0.89	0.91	0.87	0.86	0.89	0.87	0.89	0.88	0.87	0.88	0.90	0.88	0.90	0.87	0.91	0.87	0.87

Complex	Aver	1	2	3	4	5	6	7	8	9	10	11	12	13	14	15	16	17	18	19	20
1	0.66	0.00	0.70	0.62	0.65	0.63	0.64	0.64	0.69	0.66	0.77	0.60	0.61	0.78	0.67	0.61	0.68	0.64	0.69	0.67	0.69
2	0.68	0.70	0.00	0.65	0.67	0.71	0.58	0.73	0.68	0.60	0.74	0.72	0.71	0.72	0.64	0.64	0.61	0.68	0.70	0.62	0.73
3	0.67	0.62	0.65	0.00	0.66	0.67	0.60	0.71	0.70	0.69	0.73	0.63	0.62	0.82	0.66	0.68	0.61	0.71	0.71	0.59	0.72
4	0.68	0.65	0.67	0.66	0.00	0.72	0.63	0.71	0.66	0.70	0.67	0.71	0.69	0.73	0.62	0.66	0.66	0.69	0.72	0.64	0.70
5	0.69	0.63	0.71	0.67	0.72	0.00	0.73	0.67	0.64	0.70	0.70	0.70	0.68	0.84	0.61	0.68	0.69	0.69	0.80	0.61	0.74
6	0.67	0.64	0.58	0.60	0.63	0.73	0.00	0.70	0.70	0.54	0.71	0.61	0.69	0.74	0.65	0.64	0.68	0.66	0.74	0.72	0.68
7	0.68	0.64	0.73	0.71	0.71	0.67	0.70	0.00	0.76	0.58	0.67	0.62	0.70	0.69	0.68	0.79	0.66	0.61	0.63	0.77	0.66
8	0.71	0.69	0.68	0.70	0.66	0.64	0.70	0.76	0.00	0.71	0.68	0.72	0.71	0.74	0.71	0.74	0.70	0.74	0.72	0.73	0.76
9	0.66	0.66	0.60	0.69	0.70	0.70	0.54	0.58	0.71	0.00	0.70	0.63	0.69	0.74	0.68	0.70	0.60	0.57	0.64	0.70	0.68
10	0.70	0.77	0.74	0.73	0.67	0.70	0.71	0.67	0.68	0.70	0.00	0.63	0.67	0.70	0.71	0.78	0.68	0.70	0.70	0.69	0.72
11	0.65	0.60	0.72	0.63	0.71	0.70	0.61	0.62	0.72	0.63	0.63	0.00	0.57	0.73	0.74	0.68	0.68	0.66	0.60	0.62	0.60
12	0.66	0.61	0.71	0.62	0.69	0.68	0.69	0.70	0.71	0.69	0.67	0.57	0.00	0.73	0.76	0.67	0.62	0.66	0.56	0.65	0.61
13	0.74	0.78	0.72	0.82	0.73	0.84	0.74	0.69	0.74	0.74	0.70	0.73	0.73	0.00	0.73	0.72	0.70	0.73	0.72	0.76	0.80
14	0.68	0.67	0.64	0.66	0.62	0.61	0.65	0.68	0.71	0.68	0.71	0.74	0.76	0.73	0.00	0.56	0.68	0.72	0.78	0.62	0.71
15	0.68	0.61	0.64	0.68	0.66	0.68	0.64	0.79	0.74	0.70	0.78	0.68	0.67	0.72	0.56	0.00	0.69	0.71	0.72	0.59	0.66
16	0.66	0.68	0.61	0.61	0.66	0.69	0.68	0.66	0.70	0.60	0.68	0.68	0.62	0.70	0.68	0.69	0.00	0.67	0.61	0.63	0.65
17	0.68	0.64	0.68	0.71	0.69	0.69	0.66	0.61	0.74	0.57	0.70	0.66	0.66	0.73	0.72	0.71	0.67	0.00	0.63	0.71	0.63
18	0.68	0.69	0.70	0.71	0.72	0.80	0.74	0.63	0.72	0.64	0.70	0.60	0.56	0.72	0.78	0.72	0.61	0.63	0.00	0.71	0.59
19	0.67	0.67	0.62	0.59	0.64	0.61	0.72	0.77	0.73	0.70	0.69	0.62	0.65	0.76	0.62	0.59	0.63	0.71	0.71	0.00	0.68
20	0.68	0.69	0.73	0.72	0.70	0.74	0.68	0.66	0.76	0.68	0.72	0.60	0.61	0.80	0.71	0.66	0.65	0.63	0.59	0.68	0.00
Aver	0.68	0.66	0.68	0.67	0.68	0.69	0.67	0.68	0.71	0.66	0.70	0.65	0.66	0.74	0.68	0.68	0.66	0.68	0.68	0.67	0.68

AMP-PNP	Aver	1	2	3	4	5	6	7	8	9	10	11	12	13	14	15	16	17	18	19	20
1	0.68	0.00	0.68	0.66	0.71	0.69	0.70	0.72	0.69	0.59	0.64	0.78	0.72	0.71	0.62	0.74	0.67	0.82	0.59	0.62	0.60
2	0.68	0.68	0.00	0.79	0.72	0.65	0.67	0.68	0.82	0.65	0.61	0.76	0.65	0.64	0.65	0.61	0.62	0.81	0.62	0.65	0.65
3	0.70	0.66	0.79	0.00	0.67	0.76	0.59	0.68	0.58	0.72	0.70	0.67	0.80	0.69	0.79	0.67	0.78	0.68	0.73	0.69	0.62
4	0.69	0.71	0.72	0.67	0.00	0.67	0.61	0.71	0.71	0.68	0.68	0.63	0.75	0.57	0.62	0.68	0.81	0.62	0.56	0.81	0.78
5	0.69	0.69	0.65	0.76	0.67	0.00	0.70	0.65	0.72	0.77	0.71	0.66	0.59	0.74	0.68	0.63	0.66	0.70	0.69	0.70	0.71
6	0.69	0.70	0.67	0.59	0.61	0.70	0.00	0.69	0.73	0.67	0.70	0.61	0.77	0.67	0.63	0.69	0.79	0.69	0.67	0.78	0.74
7	0.69	0.72	0.68	0.68	0.71	0.65	0.69	0.00	0.74	0.79	0.58	0.64	0.67	0.69	0.73	0.62	0.62	0.72	0.71	0.71	0.77
8	0.71	0.69	0.82	0.58	0.71	0.72	0.73	0.74	0.00	0.79	0.70	0.71	0.67	0.73	0.67	0.65	0.70	0.73	0.77	0.68	0.69
9	0.70	0.59	0.65	0.72	0.68	0.77	0.67	0.79	0.79	0.00	0.62	0.77	0.77	0.69	0.67	0.78	0.68	0.70	0.65	0.71	0.58
10	0.65	0.64	0.61	0.70	0.68	0.71	0.70	0.58	0.70	0.62	0.00	0.73	0.67	0.58	0.66	0.65	0.55	0.74	0.64	0.63	0.60
11	0.70	0.78	0.76	0.67	0.63	0.66	0.61	0.64	0.71	0.77	0.73	0.00	0.76	0.70	0.66	0.64	0.71	0.56	0.71	0.82	0.81
12	0.71	0.72	0.65	0.80	0.75	0.59	0.77	0.67	0.67	0.77	0.67	0.76	0.00	0.68	0.69	0.64	0.67	0.74	0.74	0.71	0.71
13	0.68	0.71	0.64	0.69	0.57	0.74	0.67	0.69	0.73	0.69	0.58	0.70	0.68	0.00	0.60	0.64	0.72	0.62	0.59	0.80	0.77
14	0.67	0.62	0.65	0.79	0.62	0.68	0.63	0.73	0.67	0.67	0.66	0.66	0.69	0.60	0.00	0.67	0.71	0.68	0.61	0.72	0.71
15	0.66	0.74	0.61	0.67	0.68	0.63	0.69	0.62	0.65	0.78	0.65	0.64	0.64	0.64	0.67	0.00	0.65	0.64	0.65	0.66	0.68
16	0.70	0.67	0.62	0.78	0.81	0.66	0.79	0.62	0.70	0.68	0.55	0.71	0.67	0.72	0.71	0.65	0.00	0.77	0.74	0.70	0.70
17	0.71	0.82	0.81	0.68	0.62	0.70	0.69	0.72	0.73	0.70	0.74	0.56	0.74	0.62	0.68	0.64	0.77	0.00	0.69	0.83	0.80
18	0.66	0.59	0.62	0.73	0.56	0.69	0.67	0.71	0.77	0.65	0.64	0.71	0.74	0.59	0.61	0.65	0.74	0.69	0.00	0.67	0.59
19	0.71	0.62	0.65	0.69	0.81	0.70	0.78	0.71	0.68	0.71	0.63	0.82	0.71	0.80	0.72	0.66	0.70	0.83	0.67	0.00	0.60
20	0.69	0.60	0.65	0.62	0.78	0.71	0.74	0.77	0.69	0.58	0.60	0.81	0.71	0.77	0.71	0.68	0.70	0.80	0.59	0.60	0.00
Aver	0.69	0.68	0.68	0.70	0.69	0.69	0.69	0.69	0.71	0.70	0.65	0.70	0.71	0.68	0.67	0.66	0.70	0.71	0.66	0.71	0.69

**ADP**	Aver	1	2	3	4	5	6	7	8	9	10	11	12	13	14	15	16	17	18	19	20
1	0.67	0.00	0.56	0.67	0.63	0.71	0.70	0.75	0.62	0.59	0.60	0.72	0.74	0.67	0.80	0.62	0.63	0.68	0.77	0.73	0.63
2	0.69	0.56	0.00	0.66	0.58	0.70	0.75	0.71	0.69	0.62	0.68	0.64	0.77	0.73	0.89	0.65	0.60	0.72	0.79	0.71	0.67
3	0.74	0.67	0.66	0.00	0.82	0.62	0.75	0.81	0.75	0.78	0.80	0.58	0.74	0.78	0.85	0.70	0.69	0.70	0.76	0.79	0.74
4	0.70	0.63	0.58	0.82	0.00	0.79	0.68	0.75	0.63	0.59	0.74	0.72	0.72	0.70	0.77	0.66	0.64	0.70	0.70	0.78	0.73
5	0.74	0.71	0.70	0.62	0.79	0.00	0.78	0.73	0.74	0.83	0.74	0.65	0.77	0.75	0.78	0.73	0.74	0.78	0.78	0.80	0.75
6	0.70	0.70	0.75	0.75	0.68	0.78	0.00	0.64	0.64	0.77	0.81	0.73	0.69	0.75	0.69	0.64	0.61	0.63	0.64	0.67	0.79
7	0.74	0.75	0.71	0.81	0.75	0.73	0.64	0.00	0.78	0.72	0.86	0.80	0.76	0.86	0.82	0.75	0.70	0.68	0.66	0.61	0.76
8	0.70	0.62	0.69	0.75	0.63	0.74	0.64	0.78	0.00	0.69	0.75	0.75	0.63	0.73	0.84	0.70	0.64	0.70	0.63	0.70	0.75
9	0.71	0.59	0.62	0.78	0.59	0.83	0.77	0.72	0.69	0.00	0.65	0.80	0.73	0.70	0.82	0.68	0.70	0.70	0.72	0.75	0.61
10	0.72	0.60	0.68	0.80	0.74	0.74	0.81	0.86	0.75	0.65	0.00	0.75	0.70	0.54	0.70	0.76	0.76	0.73	0.80	0.82	0.58
11	0.74	0.72	0.64	0.58	0.72	0.65	0.73	0.80	0.75	0.80	0.75	0.00	0.78	0.81	0.83	0.69	0.70	0.76	0.76	0.79	0.76
12	0.71	0.74	0.77	0.74	0.72	0.77	0.69	0.76	0.63	0.73	0.70	0.78	0.00	0.68	0.63	0.67	0.71	0.68	0.67	0.78	0.68
13	0.72	0.67	0.73	0.78	0.70	0.75	0.75	0.86	0.73	0.70	0.54	0.81	0.68	0.00	0.64	0.68	0.70	0.70	0.75	0.84	0.59
14	0.78	0.80	0.89	0.85	0.77	0.78	0.69	0.82	0.84	0.82	0.70	0.83	0.63	0.64	0.00	0.72	0.83	0.79	0.81	0.83	0.68
15	0.69	0.62	0.65	0.70	0.66	0.73	0.64	0.75	0.70	0.68	0.76	0.69	0.67	0.68	0.72	0.00	0.66	0.66	0.73	0.75	0.66
16	0.69	0.63	0.60	0.69	0.64	0.74	0.61	0.70	0.64	0.70	0.76	0.70	0.71	0.70	0.83	0.66	0.00	0.69	0.74	0.67	0.76
17	0.70	0.68	0.72	0.70	0.70	0.78	0.63	0.68	0.70	0.70	0.73	0.76	0.68	0.70	0.79	0.66	0.69	0.00	0.64	0.63	0.67
18	0.73	0.77	0.79	0.76	0.70	0.78	0.64	0.66	0.63	0.72	0.80	0.76	0.67	0.75	0.81	0.73	0.74	0.64	0.00	0.75	0.73
19	0.74	0.73	0.71	0.79	0.78	0.80	0.67	0.61	0.70	0.75	0.82	0.79	0.78	0.84	0.83	0.75	0.67	0.63	0.75	0.00	0.73
20	0.70	0.63	0.67	0.74	0.73	0.75	0.79	0.76	0.75	0.61	0.58	0.76	0.68	0.59	0.68	0.66	0.76	0.67	0.73	0.73	0.00
Aver	0.72	0.67	0.69	0.74	0.70	0.74	0.70	0.74	0.70	0.71	0.72	0.74	0.71	0.72	0.78	0.69	0.69	0.70	0.73	0.74	0.70
